# Biofilm formation and potential virulence factors of *Salmonella* strains isolated from ready-to-eat shrimps

**DOI:** 10.1371/journal.pone.0204345

**Published:** 2018-09-20

**Authors:** Abeni Beshiru, Isoken H. Igbinosa, Etinosa O. Igbinosa

**Affiliations:** 1 Applied Microbial Processes & Environmental Health Research Group, Department of Microbiology, Faculty of Life Sciences, University of Benin, Benin City, Nigeria; 2 Department of Environmental Management and Toxicology, Faculty of Life Sciences, University of Benin, Benin City, Nigeria; University of Campinas, BRAZIL

## Abstract

*Salmonella* species is an important foodborne pathogen with the non-typhoidal serovars such as Enteritidis and Typhimurium as the most predominant strains. This study examines the biofilm formation, phenotypic virulence factors and cell surface characteristics of *Salmonella* strains from ready-to-eat shrimps. The ready-to-eat shrimps were obtained from open markets between November 2016 and October 2017 in Edo and Delta States, Nigeria. The occurrence of *Salmonella* strains in this study was 210/1440 (14.58%) of the ready-to-eat shrimp’s samples. The identified strains comprise of *Salmonella* Enteritidis 11, *Salmonella* Typhimurium 14 and other *Salmonella* spp. 20. The 45 identified *Salmonella* strains revealed the following virulence properties: swimming and swarming motility 45(100%); S-layer 39(86.67%); haemolytic activity 40(88.89%); lipase activity 43(95.56%); protease activity 43(95.56%); gelatinase production 43(95.56%); and DNA degrading activity 41(91.11%). The variation in the formation of biofilm-based on the diversity of *Salmonella* species was observed with higher percentage of *Salmonella* Typhimurium strains as strong biofilms producers under different environmental conditions. For surface hydrophobicity using bacterial adherence to hydrocarbons, 25(55.56%) were hydrophilic while 20(44.44%) were moderately hydrophobic from the 45 *Salmonella* isolates. Using salting aggregation test for surface hydrophobicity, all selected isolates 45(100%) was hydrophilic. Autoaggregation index for the 12 selected *Salmonella* isolates ranged from 15.2–47.2%, while the autoaggragation index for the 12 selected test bacteria ranged from 26.2–71.3%. Coaggragation between the 12 selected test bacteria and 12 *Salmonella* isolates ranged from 12.5–81.0%. The occurrence of pathogenic species of *Salmonella* from ready-to-eat shrimps could be detrimental to the consumers. Findings on the physiological conditions of biofilms formed by the foodborne pathogenic *Salmonella* and the cell surface characteristics therein are crucial for the advancement of methods for controlling *Salmonella* from ready-to-eat foods.

## Introduction

Food safety is a significant public-health concern which links farming to human health and other areas of food production [1]. Foodborne pathogenicity is the main cause of the worldwide hospitalizations and morbidity as a consequence of consumption of different foods, including seafood [2]. Currently, 31 organisms are documented as foodborne pathogens, and statistics released by the United States Centers for Disease Control and Prevention (CDC) reported that approximately 48 million foodborne illnesses occur annually in the USA alone, resulting in 128,000 hospitalizations and 3000 deaths [3–4]. Ready-to-eat shrimp products are referred to as products which consist of or which contains shrimp such as shrimp in sauce, smoked shrimp, fried shrimp, sausage and cooked/dried shrimp.

Microbiological surveillance of ready-to-eat (RTE) shrimp products provides empirical data to enlighten scientific guidance for improving the safety and quality of food. Surveillance data may be useful in explaining research priorities based on risk assessments and enlightening the development of food safety standards. Likewise, it may be an indication of direct consumer exposure the ready-to-eat shrimp products. In recent years, the demand for ready-to-eat shrimp products has consistently increased in open markets in southern Nigeria. From the farm to the consumer, processing, transportation, and storage of shrimp products possibly enables nutrient content and growth conditions to support unwanted microbial proliferation [5–7]. Due to the fact that these ready-to-eat shrimp products are not always given additional bactericidal treatment prior to consumption, the contamination of ready-to-eat shrimp products by foodborne pathogens continues to draw attention. *Listeria monocytogenes*, *Vibrio* species, *Escherichia coli*, *Campylobacter jejuni* and *Salmonella enterica* contamination accounts for the greatest number of seafood products [8–9].

In nature, significant amount of bacteria are prearranged in surface-connected communities, referred to as biofilms. Biofilm producing organisms adhere onto abiotic or biotic surfaces, and are surrounded within extracellular polymeric matrix (phospholipids, proteins, polysaccharides, and nucleic acids) which is produced by the bacteria themselves [10]. Within biofilms, bacterial cells are sheltered against different adverse environmental situations such as disinfectants, ultraviolet (UV) light radiation, osmotic changes, pH variability, dehydration, antimicrobial agents, host immune responses and metal toxicity [11]. Hence, bacterial biofilms institute an important concern for the food industry and food safety authorities where biofilm is described as a significant source of food contamination with pathogenic and spoilage microorganisms [12].

*Salmonella* species are significant causative agents of foodborne disease which has resulted in more than one million cases per year [13]. Varieties of high and low moisture foods have been ascribed as risk factors for infection in humans [14]. The biofilm-forming ability and adhesion of this disease causing agent is dependent on numerous factors such as the growth phase of the cells, growth medium, contact time, type and properties of the inert material, environmental parameters such as pH and temperature as well as the presence of organic material [15].

Previous findings have been documented and revealed that *Salmonella* can make use of multifaceted defence mechanisms to strive in harsh environmental conditions by forming compatible solutes and biofilm [10]. The protective layer produced during biofilm formation (extracellular polysaccharides and proteins) sheathes the bacterial population and affords protection as well as a measure to network with their surrounding environment [16]. It has been reported that *Salmonella* can attach to food surfaces such as grains [17], melons, cantaloupes [18], almonds [19] and tomatoes [20], with food contact surfaces [21] inclusive. Once attached, *Salmonella* can produce curli or Tafi thin fimbriae with cellulose and aggregative capabilities that are significant indicator of biofilm formation. The thin fimbriae with aggregative capabilities allow the bacterial cells to colonize and attach surfaces [16]. The capabilities of biofilm formation coupled with composition and amount of the biofilm may differ with respect to the species of *Salmonella* and their environment [12]. This could be problematic in the food industry since biofilms protect the bacteria from antibiotics, sanitizers as well as other environmental factors [10].

As a consequence of the significance of biofilm forming *Salmonella* species to public health, the multifactorial and complex phenomenon of biofilm formation has been extensively studied within the last decade under different environmental conditions [11,22–24]. However, little attention has been given to the biofilm production by *Salmonella* species from ready-to-eat shrimps with no report from Nigeria. The objective of this study was to examine biofilm formation under different conditions, surface hydrophobicity and cell surface properties of *Salmonella* strains isolated from ready-to-eat shrimps.

## Materials and methods

### Sample collection

A total of 1440 ready-to-eat shrimp samples were purchased from open markets in Delta and Edo States, Nigeria between November 2016 and October, 2017. Markets surveyed in Edo State and their coordinates include Oba market (6°20′5.52′ and 5°37′11.87), New Benin market (6.3642562 and 5.6181444), Jattu market (7.087°N and 6.287°E), Igarra market (716″59.988″N and 65″60.000″E), Ekpoma market (6°45’N and 6°08″E) and Uromi main market (3°24’E and 6°27’N). For Delta State, markets sampled include Sapele market (5°54’N 5°40’E), Ughelli main market (5°30’N 5°59’E), Ogbegonogo market Asaba (6°11’52.23”N and 6°43’42.48”E), Ashafor market, Aniocha (6°10’59.06”N and 6°31’27.72”E), Igbudu market Warri (5°31’N and 5°45’E) and main market, Oleh, Isoko (5.4043°N and 6.1951°E). Readily available ready-to-eat shrimps in the open markets include Oba market (dried and fried shrimps), New Benin market (dried and fried shrimps), Jattu market (dried and fried shrimps), Igarra market (dried and fried shrimps), Ekpoma market (dried and fried shrimps) and Uromi main market (dried and fried shrimps), Sapele market (sauced, boiled and smoked shrimps), Ughelli main market (sauced, boiled and smoked shrimps), Ogbegonogo market Asaba (sauced, boiled and smoked shrimps), Ashafor market, Aniocha (sauced, boiled and smoked shrimps), Igbudu market Warri (sauced, boiled and smoked shrimps) and main market, Oleh, Isoko (sauced, boiled and smoked shrimps).

Ten samples each were obtained from each of the respective 12 selected open markets (6 each from Delta and Edo States respectively) culminating in the 1440 ready to eat shrimp samples. Samples were obtained based on the type of ready-to-eat shrimps available with respect to the sampling location and collected using a sterile polythene bags. The sterile polythene bags were placed immediately into a cooler of ice and transported to the Applied Microbial Processes & Environmental Health Research Laboratory; Department of Microbiology, University of Benin, Benin City, Nigeria for microbiological analysis within 24 h after collection.

### Enrichment and isolation of *Salmonella* isolates

An aliquot of 1.0 mL of the stock solution (25 g of respective ready-to-eat shrimp samples homogenized in 225 mL of sterile tryptone soy broth (TSB), giving a first 10^−1^ dilution) was added into test tubes containing 9.0 mL of selenite cysteine F Broth (Lab M, Lancashire, United Kingdom) and incubated at 37°C for 24–48 h. Thereafter, streak plate technique was used via streaking directly from the turbid overnight culture of the selenite cysteine F broth on xylose lysine deoxycholate (XLD) agar (Lab M, Lancashire, United Kingdom) and incubated at 37°C for 24–48 h [25]. A typical red colony with black centres after incubation were characteristically described tentatively as *Salmonella* isolate and sub-cultured on Hektoen enteric agar (HEA) (Lab M, Lancashire, United Kingdom) and incubated at 37°C for 24–48 h. After incubation, green colonies with or without black centres were repeatedly purified on Nutrient agar (Lab M, Lancashire, United Kingdom) for at 37°C for 24–48 h and presumptively identified as *Salmonella* isolate. The purified isolates were stored in Nutrient agar slants at 4°C until ready for further use.

### Morphological and biochemical identification of *Salmonella* species

All *Salmonella* isolates were subjected to cultural (characteristics on HEA and XLD agar), morphological (Gram reaction 3% KOH test and microscopy) and biochemical (catalase, oxidase, indole, citrate, and sugar fermentation test) characterization. Analytical Profile Index 20E (API 20E) was used to identify *Salmonella* isolates in accordance with the manufacturer’s instructions (BioMerieux, Marcy-l'Étoile, France). Final biochemical identification was carried out via API lab plus software (bioMerieux, Marcy l’Etoile, France). A classification of the identified isolates based on the API profile as good, very good and excellent identification outputs were considered prior to molecular identification of the isolates.

### Genomic deoxyribonucleic acid (gDNA) extraction protocol

Genomic DNA from *Salmonella* isolates were extracted by using the standard boiling method described previously by Igbinosa et al. [26] with slight modification. *Salmonella* isolates from the initial pure culture were used to prepare suspension in 5.0 mL of tryptone soy broth (TSB) (Merck, Darmstadt, Germany) and incubated overnight at 37 °C for 18–24 h. Then 100 μL of the turbid suspension was diluted with 100 μL of sterilized deionized water in a 2.0 mL eppendorf tube. Immediately, the cell mixture followed a lysing procedure using a dry bath (MK200-2, Shanghai, China) at 100 °C for 15 min and subjected to centrifugation using a mini centrifuge (Mini 14k, Zhuhai, Guangdong, China) at 14,500 r/min for 15 min. The cell debris was carefully separated while the supernatant was used as the gDNA template.

### Polymerase chain reaction amplification procedure

All reactions were performed in 25.0 μL volume of reaction (10 × Buffer 2.5 μL; MgCl_2_ 1.0 μL; dNTP-Mix 3.0 μL; Taq polymerase 0.2 μL; reversed primer 1.25 μL; forward primer 1.25 μL; sterile double distilled H_2_0 10.8 μL and gDNA 5.0 μL). All primers used for the confirmation of *Salmonella* isolates are shown in Table 1. Using a Peltier-based Thermal Cycler (BioSeparation System, Shanxi, China) and primers previously described [27–28], the reaction was performed using an initial denaturation at 95°C for 10 min; 35 cycles of denaturation at 94°C for 60s, primer annealing and extension at 72°C for 90s; final extension at 72°C for 10 min. *Salmonella enterica* serovar Typhymurium ATCC 14028, *Salmonella* Enteritidis ATCC 13076, were used as positive controls. Deionized water was used as a negative control for each test procedure. Electrophoresis of the PCR amplified products was carried out with 1.5% agarose gel (CLS-AG100, Warwickshire, United Kingdom) in 0.5× TAE buffer (pH 8.5, 20 mM Na acetate, 40 mM Tris-HCl, 1 mM EDTA) and allowed to run for 1 h at 100 V. Thereafter, the gels are visualized under a UV transilluminator (EBOX VX5, Vilber Lourmat, France).

**Table 1:** Primers used in the identification of *Salmonella* species.

Target species	Primer name	Sequence (5' to 3')	Target gene	Annealing condition	Amplicon size (bp)	Reference
*Salmonella* genus	S. 16S Rdnaf	TGTTGTGGTTAATAACCGCA	16S rDNA gene	56 °C for 60 s	574	Ziemer and Steadham [17]
	S. 16S Rdnar	CACAAATCCATCTCTGGA				
*Salmonella* Enteritidis	ENT-F	AAATGTGTTTTATCTGATGCAAGAGG	*Ent*	60°C for 90 s	299	Saeki et al.[18]
	ENT-R	GTTCGTTCTTCTGGTACTTACGATGAC				
*Salmonella* Typhimurium	STM4492-F	ACAGCT TGGCCTACGCGAG	*Stm*4492	60°C for 90 s	759	Saeki et al.[18]
	STM4492-R	AGCAACCGTTCGGCCTGAC				

### Physiological characteristics of extracellular virulence factors for *Salmonella* species

Colonies grown on tryptone soy agar (TSA) (Merck, Darmstadt, Germany) were re-suspended in 3.0 mL of TSB. The turbidity of this suspension was adjusted to 0.5 McFarland standards, which is the equivalent of 10^8^ cells/mL. A 5.0 mL sample of this suspension was inoculated on sheep blood agar plate and incubated for 24–48 h at 37 °C. Haemolytic activity was indicated by clear colourless zones surrounding the colonies indicating that there have been lyses of the red blood cells. For lipase activity, 5.0 mL sample of the suspension was inoculated on TSA and incubated for 24–48 h at 37 °C. When a bacterium produces lipase on the agar, a clear halo surrounds the areas where the lipase-producing bacterium has grown. For protease activity, 5.0 mL sample of the suspension was inoculated on TSA plates supplemented with 1% casein and incubated for 24–48 h at 37 °C. Clear zone as a result of casein hydrolysis was considered a positive result. For gelatinase production, 5.0 mL sample of the suspension was inoculated on gelatin medium and incubated for 24–48 h at 37 °C. Zones of clearance in the media indicated the proliferation of gelatin-liquefying microorganisms. For DNA degrading activity, 5.0 mL sample of the suspension was inoculated on DNase agar plates and incubated for 24–48 h at 37 °C. When DNA is broken down, it results in the release of methyl green which turns the medium colourless around the test organism. *Salmonella* isolates were assessed for motility characteristics, swarming (1% tryptone, 0.6% agar, 0.5% NaCl) and swimming (1% tryptone, 0.25% agar, 0.5% NaCl), as described by Altarriba et al. [29]. The presence of surface-layer (S-layer) was assessed by repeatedly streaking cultures on TSA plates, enhanced with 0.1 mg/mL Coomassie brilliant blue R 250 (Merck, Darmstadt Germany) as described by Bernoth [30].

### Characterization of biofilm formation

All *Salmonella* isolates were cultivated overnight in TSB (Merck, Darmstadt, Germany) and centrifuged for 2 min at 12 000 rpm. The cell pellets were washed thrice and re-suspended in phosphate-buffered saline (PBS) pH 7.2 to cell densities equivalent to 0.5 McFarland standard [31]. To determine the bacterial adherence to the microtitre plate, sterile 96-well polystyrene microtitre plates (Nest Biotech Co., Ltd, Jiangsu Province, China) with each well of the microtitre plate filled with a 90 μL TSB (more enriched with the following constituent: 2.5 g/L dipotassium hydrogen phosphate; 5.0 g/L soy peptone; 15.0 g/L tryptone; 5.0 g/L sodium chloride; 2.5 g/L dextrose) or enriched anacker and ordal broth (EAOB) (less enriched compared to TSB with the following constituent: 0.2 g/L beef extract; 5.0 g/L tryptone; 0.2 g/L sodium acetate; 0.5 g/L yeast extract) and inoculated with 10 μL of standardized *Salmonella* species cell suspensions in triplicate [32]. Negative control wells which contain only TSB, PBS and EAOB were added in each assay, while suspension of *Salmonella* Enteritidis ATCC 13076 was included as a positive control. Respective plates were placed on a platform shaker (simulate dynamic condition) and normal incubator (static condition), and incubated aerobically for 24 h at 21 °C, 30 °C and 37 °C. Contents of respective wells were thereafter aspirated, washed thrice with sterile 250 μL PBS with the cells remaining fixed for 15 min with 200 μL of methanol (Fisher Scientific, New Hampshire, United States). Afterwards, air-dried wells were stained for 5 min with 150 μL of crystal violet dye (2%) (Fisher Scientific, New Hampshire, United States). Adherent cells that were dye bound were re-solubilized using 150 μL of 33% (v/v) glacial acetic acid (Fisher Scientific, New Hampshire, United States). Thereafter, the optical density (OD) of each well was determined at 595 nm using an automated microtitre plate reader (Synergy MxBiotekR, Winooski, Vermont, USA). All biological assays were conducted in triplicate. Biofilm formation was determined as described previously by Stepanovic et al. [32]. Three standard deviations above the mean OD of the negative control for the microtitre plate test were defined as the cut-off optical density (OD) (ODc). Isolates were classified as follows: (4 × OD_C_) < OD = strongly adherent, (2 × OD_C_) < OD ≤ (4 × OD_C_) = moderately adherent, OD_C_ < OD ≤ (2 × OD_C_) = weakly adherent and OD ≤ OD_C_ = non-adherent [32].

The relative biofilm-forming capacity for 50 selected *Salmonella* isolate was reported relative to the mean value of all isolates as follows:

$$\frac{\left[A_{X}-A_{0}\right]}{\left[\sum_{n=1}^{50}(A_{n}-A_{0}]/50\right)}$$

Where A_0_ is equivalent to the absorbance for un-inoculated growth medium, A_x_ is equivalent to the absorbance at 595 nm for isolate x [33].

### Bacterial hydrophobicity bioassay

Bacterial surface hydrophobicity was assayed for adopting the bacterial adherence to hydrocarbons (BATH) protocol, using xylene (BDH, VWR International, Leicestershire, United Kingdom) as the chosen hydrocarbon [34]. Species of *Salmonella* grown in TSB at 37 °C were recovered during the log growth phase, washed thrice and re-suspended in sterilized 0.1 M phosphate buffer with pH 7.0 to OD of 0.8 via wavelength of 550 nm equivalent to A_0_ of 10^6^ CFU/mL. Three (3.0) mL of bacterial suspensions were gently placed in glass tubes aseptically with 400 μL of xylene, maintained in a water bath for 10 min at 25 °C and agitated. After a phase of separation for 15 min, the bottom aqueous phase was extracted, with its OD_550_ assayed (A_1_). *Salmonella* strains were regarded as hydrophilic when values were <20%, moderately hydrophobic when values were in the range of 20–50% and strongly hydrophobic when values were >50% [35]. All biological assays were carried out in triplicate on three independent experiments. *Salmonella* Enteritidis ATCC 13076 was used as a positive control while negative control used was PBS.

A twenty-four (24) h incubated TSB cultures were recovered, washed three times and re-suspended in PBS (pH 7.2). For the salting aggregation test (SAT) modified assay, respective isolates of *Salmonella* were aggregated by salting out via the combination of 25 μL (2 × 10^6^ bacteria), with 25 μL of methylene blue-containing ammonium sulphate [(NH_4_)_2_SO_4_] at respective preceding concentrations (0.1, 0.2, 0.5, 1, 1.5, 2, 2.5, 3 and 4 mol/L) on clean grease-free microscope slides [31] accompanied by vortex for 4 min at room temperature. The least final concentration of (NH_4_)_2_SO_4_ where aggregation occurred was reported as the SAT value and designated as follows: >1.0 mol/L = hydrophilic, 0.1–1.0 mol/L = hydrophobic and <0.1 mol/L = highly hydrophobic [36]. All biological assays were carried out in triplicate on three independent experiments; (NH_4_)_2_SO_4_ concentrations on clean grease-free slides were used as negative controls.

### Coaggregation and autoaggregation bioassays

For self-aggregation (autoaggregation) and coaggregation bioassays, bacteria were cultivated in 20 mL TSB, recovered after 36 h, washed and re-suspended in sterilized distilled H_2_O to an optical density (OD) of 0.3 at 660 nm wavelength. The percentage of self-aggregation for the *Salmonella* isolates was measured by transferring 1.0 mL of bacterial suspension (2 × 10^6^ bacteria) to a 2.0 mL sterilized plastic cuvette and OD measured 60 min [37] using a T80 UV/VIS spectrophotometer (Alma Park, Wibtoft, Leicestershire, England) at 660 nm wavelength. The level of self-aggregation was reported as the percentage reduction of OD after 60 min with the equation that follows:

$$Percentage\;autoaggregation=\frac{{OD}_{0}-{OD}_{60}}{{OD}_{0}}\times 100$$

OD_0_ denotes the previous OD of the organism assayed. Sixty minutes (60 min) later; the cell suspension (with which OD_0_ was obtained) was centrifuged for 2 min at 2000 rpm with the OD of the supernatant was determined (OD_60_) [37]. All biological assays were carried out in triplicate on three independent experiments [37].

Twelve *Salmonella* species with moderate and strong biofilm forming potential were assayed for their capabilities to coaggregate with the following test bacterial as partner strains, *Salmonella* Enteritidis ATCC 13076, *Aeromonas hydrophila* ATCC 7966, *Acinetobacter baumannii* ATCC 19606, *Salmonella enterica* serovar Typhymurium ATCC 14028, *Staphylococcus aureus* ATCC 25823, *Escherichia coli* ATCC 29214, *Shigella flexneri* ATCC 120222, *Listeria innocua* ATCC 33090, *Pseudomonas aeruginosa* ATCC 29853, *Listeria monocytogenes* ATCC 7644, *Pseudomonas putida* ATCC 15175, and *Bacillus cereus* ATCC 14579. The level of coaggregation was estimated by the readings obtained from the OD via paired isolate suspensions (500 μL of each test bacteria and *Salmonella* strain assayed). The cell combination was centrifuged for 2 min at 2000 rpm, with the OD from 600 μL of the supernatant determined at 660 nm wavelength [37]. The coaggregation rate of paired isolates was quantified using the equation that follows:

$$Percentage\;coaggregation=\frac{{OD}_{Tot}-{OD}_{s}}{{OD}_{Tot}}\times 100$$

OD_Tot_ data denotes the initial OD obtained instantly following when the strains of interest were paired, while ODs denotes the OD of the supernatant, following centrifugation after 60 min [37]. All biological assays were carried out in triplicate on three independent experiments.

### Statistical analysis

All data in this study were statistically analysed using the Statistical Package (SPSS) version 21.0 and Microsoft Excel 2013. Biofilm characterization and *Salmonella* hydrophobicity were assayed using descriptive statistics and expressed as mean ± standard deviation. Virulence factors and cell surface characteristics were expressed in percentage and analysed using One Sample T-test. Biofilm characterization was analysed using one way analysis of variance and Duncan multiple range test to indicate difference between mean. Correlation analysis between the biofilm formation and extracellular virulence factors were analysed. The *p*-value < 0.05 were reported significant.

## Results

### Isolation and detection of *Salmonella* isolates from ready-to-eat shrimp

This study revealed, 210/1440 (14.58%) of the ready-to-eat shrimp samples were positive for *Salmonella* species. All the tentatively 210 *Salmonella* isolates were characterized with the culture-based using Gram-reaction with 3% KOH test, oxidase, urease reactions, indole and motility tests. The *Salmonella* isolates that appear negative for oxidase, urease, indole and Gram-negative rods were selected as presumptive *Salmonella*. Only 67 *Salmonella* isolates, were positive using culture-based approach, Analytical profile index (API) were further employed to confirm the biochemical and enzymatic reactions of the isolates and revealed (49) *Salmonella* isolates. From the 49 *Salmonella* isolates positive from the API test, *Salmonella* genus-specific primer was only positive for 45 isolates. This was further identified using the species-specific primer that target *Salmonella* Enteritidis 11, *Salmonella* Typhimurium 14 and other *Salmonella* spp. 20.

### Phenotypic characterization of *Salmonella* species

**Fig 1:**
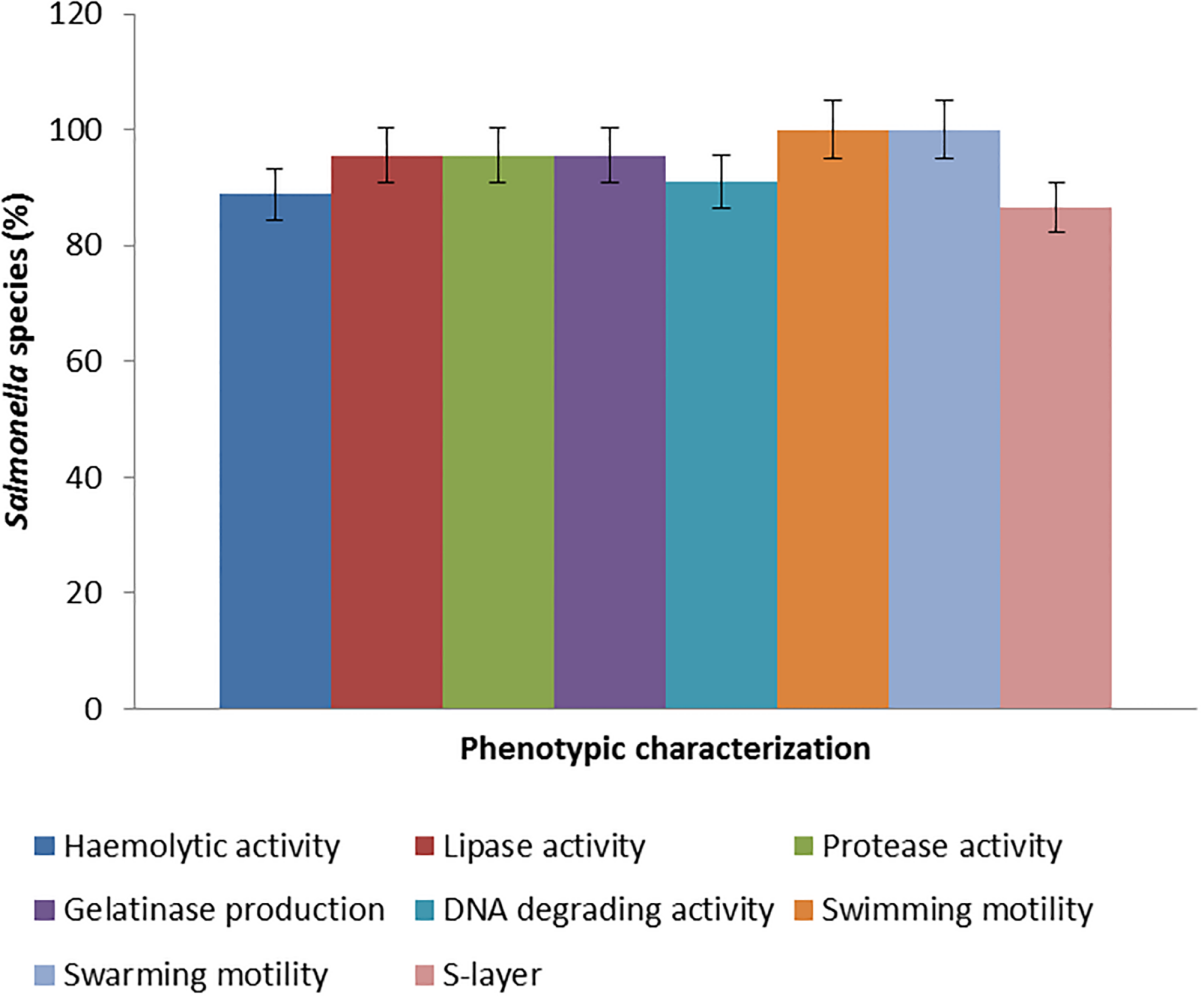
Total phenotypic characteristics of the *Salmonella* species.

Phenotypic virulence profile of the *Salmonella* species from ready-to-eat shrimps is shown in Table 2. All *Salmonella* isolates 45/45 (100%) in this study displayed swimming and swarming motility. However, for *Salmonella* Enteritidis, 10/11 (90.9%) showed haemolytic activity, 9/11 (86.7%) revealed the presence of S-layer, 11/11 (100%) displayed lipase activity, 11/11 (100%) revealed protease activity, 10/11 (90.9%) showed gelatinase production, and 11/11 (100%) portrayed DNA degrading activity. For *Salmonella* Typhimurium, 12/14 (90%) revealed the presence of S-layer, 14/14 (100%) showed haemolytic activity, 14/14 (100%) revealed lipase activity, 14/14 (100%) portrayed protease activity, 14/14 (100%) displayed gelatinase production and 12/14 (85.7%) revealed DNA degrading activity. For other *Salmonella* spp., 18/20 (91.4%) revealed the presence of S-layer, 16/20 (80%) revealed haemolytic activity, 18/20 (90%) showed lipase activity, 18/20 (90%) displayed protease activity, 19/20 (95%) revealed gelatinase production, and 18/20 (90%) portrayed DNA degrading activity. In total, 40/45 (88.9%) displayed haemolytic activity, 43/45 (95.6%) revealed lipase activity, 43/45 (95.6%) portrayed protease activity, 43/45 (95.56%) showed gelatinase production, 39/45 (86.67%) displayed the presence of S-layer and 41/45 (91.11%) revealed DNA degrading activity (Fig 1). *Salmonella* swarming motility positively correlates biofilm formation (*r* = 0.965; *p* = 0.01) and extracellular virulence factor production (*r* = 0.912; *p* = 0.01). A negative correlation of protease on biofilm formation exist (*r* = -0.722; *p* = 0.05). Gelatinase production positively correlates biofilm formation (*r* = 0.710; *p* = 0.05). There was no correlation of haemolysin on formation of biofilm (*r* = 0.448; *p* = 0.05). Positive correlation of lipolytic activity on biofilm formation (*r* = 0.825; *p* = 0.01) exist. S-layer negatively correlates biofilm formation (*r* = -0.801; *p* = 0.01).

**Table 2:** Phenotypic characterization of *Salmonella* species from ready-to-eat shrimps.

*Salmonella* species	Haemolytic activity	Lipase activity	Protease activity	Gelatinase production	DNA degrading activity	Swimming motility	Swarming motility	S-layer
*Salmonella* Enteritidis (*n* = 11)	10(90.9)	11(100)	11(100)	10(90.9)	11(100)	11(100)	11(100)	9(86.7)
*Salmonella* Typhimurium (*n* = 14)	14(100)	14(100)	14(100)	14(100)	12(85.7)	14(100)	14(100)	12(90)
Other *Salmonella* spp. (*n* = 20)	16(80)	18(90)	18(90)	19(95)	18(90)	20(100)	20(100)	18(91.4)

### Biofilm characterization of *Salmonella* species

Biofilm characterization of *Salmonella* species is presented in Table 3. Majority of the isolates adhered strongly at 30°C. Biofilm was assessed at 21°C, 30°C, and 37°C, in enriched anacker and ordal broth (EAOB) and tryptone soy broth (TSB) as well as under static or dynamic conditions. For *Salmonella* Enteritidis, at 30°C EAOB dynamic, OD ranged from 0.07±0.01–0.53±0.00. At 30°C EAOB static, OD ranged from 0.08±0.02–0.56±0.02. At 30°C TSB dynamic, OD ranged from 0.10±0.00–0.57±0.05. At 30°C TSB static, OD ranged from 0.12±0.00–0.63±0.11. In total, 10/11 (90.91%) were biofilm producers at 30°C EAOB dynamic, 10/11 (90.91%) were biofilm producers at 30°C EAOB static, 9/11 (81.82%) were biofilm producers at 30°C TSB dynamic, 9/11 (81.82%) were biofilm producers at 30°C TSB static.

**Table 3:** Characterization of biofilm formation by *Salmonella* species.

*Salmonella* species	Parameters	Non adherent		Weak		Moderate		Strong		Total	
			Average		Average		Average		Average		Average
		Number (%)	OD ± SD	Number (%)	OD ± SD	Number (%)	OD ± SD	Number (%)	OD ± SD	Number (%)	OD ± SD
*Salmonella* Enteritidis (*n* = 11)	21°C EAOB dynamic	-	-	1 (9.09)	0.14±0.01^a^	3 (27.27)	0.25±0.03^a^	7 (63.63)	0.37±0.03^a^	11 (100)	0.25±0.13^a^
	21°C EAOB static	-	-	1 (9.09)	0.15±0.02^a^	3 (27.27)	0.27±0.02^b^	7 (63.63)	0.38±0.12^a^	11 (100)	0.27±0.11^a^
	21°C TSB dynamic	1 (9.09)	0.06±0.00^a^	2 (18.18)	0.17±0.01^b^	3 (27.27)	0.27±0.01^b^	5 (45.45)	0.38±0.04^a^	10 (90.91)	0.29±0.07^b^
	21°C TSB static	1 (9.09)	0.08±0.01^c^	2 (18.18)	0.18±0.00^b^	4 (36.36)	0.30±0.01^c^	4 (36.36)	0.41±0.03^b^	10 (90.91)	0.32±0.05^b^
	30°C EAOB dynamic	1 (9.09)	0.07±0.01^b^	2 (18.18)	0.22±0.01^c^	4 (36.36)	0.35±0.03^d^	4 (36.36)	0.53±0.00^d^	10 (90.91)	0.39±0.15^c^
	30°C EAOB static	1 (9.09)	0.08±0.02^c^	2 (18.18)	0.25±0.03^c^	4 (36.36)	0.38±0.03^d^	4 (36.36)	0.56±0.02^d^	10 (90.91)	0.42±0.04^d^
	30°C TSB dynamic	2 (18.18)	0.10±0.00^d^	2 (18.18)	0.27±0.03^d^	4 (36.36)	0.39±0.05^d^	3 (27.27)	0.57±0.05^d^	9 (81.82)	0.44±0.02^d^
	30°C TSB static	2 (18.18)	0.12±0.00^d^	3 (27.27)	0.26±0.02^d^	5 (45.46)	0.42±0.02^d^	1 (9.09)	0.63±0.11^d^	9 (81.82)	0.48±0.13^d^
	37°C EAOB dynamic	1 (9.09)	0.05±0.01^a^	2 (18.18)	0.17±0.00^d^	3 (27.27)	0.25±0.02^a^	5 (45.45)	0.36±0.03^a^	10 (90.91)	0.28±0.07^a^
	37°C EAOB static	1 (9.09)	0.06±0.00^a^	2 (18.18)	0.18±0.02^b^	3 (27.27)	0.28±0.05^b^	5 (45.45)	0.39±0.10^a^	10 (90.91)	0.30±0.03^b^
	37°C TSB dynamic	1 (9.09)	0.08±0.02^c^	2 (18.18)	0.18±0.02^b^	4 (36.36)	0.28±0.04^b^	4 (36.36)	0.38±0.02^a^	10 (90.91)	0.31±0.12^b^
	37°C TSB static	1 (9.09)	0.08±0.02^c^	2 (18.18)	0.20±0.01^c^	4 (36.36)	0.29±0.05^b^	4 (36.36)	0.42±0.02^b^	10 (90.91)	0.33±0.04^b^
*Salmonella* Typhimurium (*n* = 14)	21°C EAOB dynamic	-	-	1 (7.14)	0.15±0.02^a^	2 (14.29)	0.27±0.02^b^	11 (78.57)	0.35±0.13^a^	14 (100)	0.26±0.13^a^
	21°C EAOB static	-	-	1 (7.14)	0.16±0.02^b^	2 (14.29)	0.28±0.01^b^	11 (78.57)	0.36±0.12^a^	14 (100)	0.27±0.03^a^
	21°C TSB dynamic	-	-	1 (7.14)	0.16±0.05^b^	2 (14.29)	0.29±0.02^b^	11 (78.57)	0.36±0.07^a^	14 (100)	0.27±0.13^a^
	21°C TSB static	1 (7.14)	0.06±0.03^a^	1 (7.14)	0.18±0.04^b^	3 (21.43)	0.29±0.04^b^	9 (64.29)	0.37±0.05^a^	13 (92.86)	0.30±0.12^b^
	30°C EAOB dynamic	1 (7.14)	0.07±0.02^b^	1 (7.14)	0.21±0.05^c^	3 (21.43)	0.35±0.02^d^	9 (64.29)	0.43±0.05^c^	13 (92.86)	0.35±0.04^c^
	30°C EAOB static	1 (7.14)	0.08±0.02^c^	1 (7.14)	0.22±0.01^c^	4 (28.57)	0.36±0.02^d^	8 (57.14)	0.47±0.03^c^	13 (92.86)	0.38±0.02^c^
	30°C TSB dynamic	1 (7.14)	0.08±0.02^c^	2 (14.29)	0.25±0.01^c^	3 (21.43)	0.37±0.01^d^	8 (57.14)	0.62±0.01^d^	13 (92.86)	0.44±0.04^d^
	30°C TSB static	1 (7.14)	0.10±0.01^d^	2 (14.29)	0.26±0.01^d^	4 (28.57)	0.39±0.01^d^	7 (50)	0.81±0.11^e^	13 (92.86)	0.52±0.14^e^
	37°C EAOB dynamic	1 (7.14)	0.06±0.00^a^	1 (7.14)	0.15±0.03^a^	2 (14.29)	0.23±0.04^a^	10 (71.45)	0.36±0.13^a^	13 (92.86)	0.27±0.23^a^
	37°C EAOB static	1 (7.14)	0.07±0.00^b^	1 (7.14)	0.16±0.01^b^	3 (21.43)	0.25±0.03^a^	9 (64.29)	0.39±0.12^a^	13 (92.86)	0.29±0.02^b^
	37°C TSB dynamic	1 (7.14)	0.07±0.01^b^	1 (7.14)	0.16±0.01^b^	2 (14.29)	0.24±0.03^a^	10 (71.45)	0.38±0.08^a^	13 (92.86)	0.28±0.01^a^
	37°C TSB static	1 (7.14)	0.08±0.00^c^	1 (7.14)	0.17±0.01^b^	4 (28.57)	0.26±0.01^a^	8 (57.14)	0.41±0.04^b^	13 (92.86)	0.31±0.13^b^
Other *Salmonella* spp. (*n* = 20)	21°C EAOB dynamic	-	-	1 (5.00)	0.14±0.01^a^	7 (35.00)	0.29±0.02^b^	12 (60.00)	0.36±0.13^a^	20 (100)	0.26±0.13^a^
	21°C EAOB static	-	-	1 (5.00)	0.16±0.00^b^	7 (35.00)	0.30±0.01^c^	12 (60.00)	0.38±0.07^a^	20 (100)	0.28±0.11^a^
	21°C TSB dynamic	1 (5.00)	0.06±0.01^a^	2 (10.00)	0.17±0.01^b^	7 (35.00)	0.32±0.01^c^	10 (50.00)	0.37±0.05^a^	19 (95.00)	0.31±0.12^b^
	21°C TSB static	1 (5.00)	0.08±0.03^c^	2 (10.00)	0.19±0.02^b^	8 (40.00)	0.32±0.01^c^	9 (45.00)	0.41±0.13^b^	19 (95.00)	0.33±0.07^b^
	30°C EAOB dynamic	1 (5.00)	0.07±0.02^b^	2 (10.00)	0.22±0.04^c^	12 (60.00)	0.41±0.03^d^	5 (25.00)	0.52±0.02^d^	19 (95.00)	0.41±0.05^c^
	30°C EAOB static	1 (5.00)	0.09±0.02^d^	2 (10.00)	0.23±0.01^c^	13 (65.00)	0.43±0.02^d^	4 (20.00)	0.55±0.04^d^	19 (95.00)	0.43±0.10^d^
	30°C TSB dynamic	1 (5.00)	0.10±0.01^d^	2 (10.00)	0.25±0.01^c^	13 (65.00)	0.44±0.02^d^	4 (20.00)	0.61±0.05^d^	19 (95.00)	0.47±0.06^d^
	30°C TSB static	2 (10.00)	0.12±0.03^d^	3 (15.00)	0.26±0.02^d^	14 (70.00)	0.45±0.03^d^	1 (5.00)	0.70±0.12^e^	18 (90.00)	0.51±0.05^e^
	37°C EAOB dynamic	1 (5.00)	0.06±0.02^a^	1 (5.00)	0.14±0.02^a^	6 (30.00)	0.25±0.01^a^	12 (60.00)	0.35±0.04^a^	19 (95.00)	0.27±0.07^a^
	37°C EAOB static	1 (5.00)	0.07±0.02^b^	1 (5.00)	0.15±0.03^a^	6 (30.00)	0.26±0.03^a^	12 (60.00)	0.37±0.02^a^	19 (95.00)	0.28±0.05^a^
	37°C TSB dynamic	1 (5.00)	0.07±0.01^b^	1 (5.00)	0.15±0.01^a^	7 (35.00)	0.26±0.02^a^	11 (55.00)	0.38±0.13^a^	19 (95.00)	0.29±0.13^a^
	37°C TSB static	1 (5.00)	0.08±0.01^c^	1 (5.00)	0.17±0.01^b^	7 (35.00)	0.27±0.11^b^	11 (55.00)	0.39±0.06^a^	19 (95.00)	0.30±0.06^b^
	*p*-value		0.001		0.000		0.000		0.000		0.000

Data are the mean of independent experiments in triplicate ± standard deviation following growth in minimal (EAOB) and rich (TSB) media at 21 °C, 30 °C and 37 °C under static and dynamic conditions respectively. OD values with different alphabets across column show significant difference (*p*<0.05).

For *Salmonella* Typhimurium, at 30°C EAOB dynamic, OD ranged from 0.07±0.02–0.43±0.05. At 30°C EAOB static, OD ranged from 0.08±0.02–0.47±0.03 At 30°C TSB dynamic, OD ranged from 0.08±0.02–0.62±0.01. At 30°C TSB static, OD ranged from 0.10±0.01–0.81±0.11. In total, 13/14 (92.86%) were biofilm producers at 30°C EAOB dynamic, 30°C EAOB static, 30°C TSB dynamic, and 30°C TSB static.

For other *Salmonella* species, at 30°C EAOB dynamic, OD ranged from 0.07±0.02–0.52±0.02. At 30°C EAOB static, OD ranged from 0.09±0.02–0.55±0.04. At 30°C TSB dynamic, OD ranged from 0.10±0.01–0.61±0.05. At 30°C TSB static, OD ranged from 0.12±0.03–0.70±0.12. In total, 19/20 (95%) were biofilm producers at 30°C EAOB dynamic, 30°C EAOB static and 30°C TSB dynamic, while 18/20 (90%) were biofilm producers at 30°C TSB static.

The variation in the formation of biofilm-based on the diversity of *Salmonella* species was observed with higher percentage of *Salmonella* Typhimurium strains as strong biofilms producers at 21, 30 and 37 °C under nutrient-deprived and nutrient-rich medium, with either static or dynamic conditions, with the exemption of 37°C TSB static. Other *Salmonella* spp. was the strongest biofilm producers at 37 °C in nutrient-rich medium and in static conditions.

### Biofilm forming capacity, surface hydrophobicity determinations of the *Salmonella* species

Biofilm forming capacity, surface hydrophobicity determinations of the *Salmonella* species is presented in Table 4. Biofilm producing *Salmonella* isolates with an OD range of 0.25±0.01–0.59±0.01 for EAOB and OD 0.29±0.06–0.66±0.03 for TSB at OD 595 nm were screened for their relative biofilm-forming capacity and surface hydrophobicity. For the relative biofilm-forming capacity, OD ranges from 0.65±0.02–1.66±0.02 for EAOB and from 0.63±0.13–1.60±0.02 on TSB. For surface hydrophobicity using BATH, 25/45 (55.56%) were hydrophilic as values were < 20% while 20/45 (44.44%) were moderately hydrophobic as values were in the range of 20–50%. Using SAT (NH_4_)_2_SO_4_, all isolates 45/45 (100%) were classified as hydrophilic with values > 1.0 mol/L. There was no correlation on bacterial hydrophobicity and biofilm formation (*r* = 0.319; *p* = 0.05).

**Table 4:** Biofilm formation, relative biofilm forming capacity, surface hydrophobicity determinations of *Salmonella* species.

Isolate code	*Salmonella* species	Biofilm formation (OD 595 nm)		Relative biofilm-forming capacity		Surface hydrophobicity	
		EAOB	TSB	EAOB	TSB	BATH (%)	SAT (NH_4_)_2_SO_4_
S009	*Salmonella* Typhimurium	0.27±0.01	0.31±0.03	0.71±0.02	0.68±0.07	12.10±0.01	2.0±0.0
S023	Other *Salmonella* sp.	0.56±0.02	0.69±0.01	1.57±0.06	1.60±0.02	20.22±0.01	2.5±0.0
S055	*Salmonella* Enteritidis	0.25±0.01	0.31±0.02	0.65±0.02	0.68±0.04	12.33±0.02	2.0±0.0
S067	*Salmonella* Typhimurium	0.36±0.01	0.43±0.04	0.97±0.02	0.97±0.12	18.12±0.02	2.0±0.0
S078	Other *Salmonella* sp.	0.51±0.02	0.65±0.01	1.42±0.04	1.51±0.02	22.15±0.01	2.5±0.0
S083	Other *Salmonella* sp.	0.43±0.01	0.51±0.07	1.19±0.02	1.17±0.17	23.03±0.02	2.0±0.0
S090	*Salmonella* Enteritidis	0.28±0.05	0.33±0.09	0.74±0.14	0.73±0.21	14.12±0.02	2.5±0.0
S125	Other *Salmonella* sp.	0.29±0.07	0.35±0.02	0.77±0.23	0.78±0.05	10.43±0.01	2.0±0.0
S133	*Salmonella* Enteritidis	0.32±0.09	0.39±0.02	0.86±0.29	0.87±0.04	13.56±0.01	2.0±0.0
S142	Other *Salmonella* sp.	0.28±0.02	0.34±0.01	0.74±0.07	0.75±0.03	17.64±0.01	2.5±0.27
S157	Other *Salmonella* sp.	0.46±0.01	0.57±0.01	1.27±0.03	1.31±0.03	20.27±0.03	4.0±0.0
S164	*Salmonella* Typhimurium	0.29±0.03	0.34±0.01	0.77±0.05	0.75±0.02	9.55±0.01	2.0±0.0
S170	Other *Salmonella* sp.	0.59±0.01	0.66±0.03	1.66±0.02	1.53±0.06	21.28±0.03	2.0±0.0
S181	*Salmonella* Enteritidis	0.42±0.01	0.49±0.03	1.16±0.02	1.12±0.09	20.69±0.02	2.5±0.27
S196	Other *Salmonella* sp.	0.46±0.01	0.59±0.01	1.27±0.03	1.36±0.04	24.16±0.02	2.0±0.0
S203	*Salmonella* Enteritidis	0.51±0.03	0.60±0.02	1.42±0.07	1.38±0.08	20.17±0.03	3.0±0.0
S211	Other *Salmonella* sp.	0.45±0.02	0.49±0.05	1.24±0.05	1.12±0.15	22.19±0.02	3.0±0.0
S220	*Salmonella* Typhimurium	0.35±0.01	0.43±0.02	0.95±0.02	0.97±0.03	16.24±0.01	2.0±0.0
S231	Other *Salmonella* sp.	0.47±0.01	0.56±0.05	1.30±0.02	1.29±0.07	19.87±0.01	3.0±0.0
S242	Other *Salmonella* sp.	0.32±0.04	0.46±0.08	0.86±0.08	1.04±0.11	18.99±0.01	2.5±0.0
S257	*Salmonella* Typhimurium	0.39±0.05	0.45±0.06	1.07±0.09	1.02±0.20	20.03±0.02	2.5±0.0
S261	Other *Salmonella* sp.	0.29±0.01	0.33±0.02	0.77±0.02	0.73±0.07	14.35±0.01	2.0±0.0
S278	*Salmonella* Typhimurium	0.57±0.01	0.63±0.03	1.60±0.02	1.46±0.09	20.19±0.01	2.0±0.0
S289	Other *Salmonella* sp.	0.41±0.02	0.46±0.02	1.13±0.04	1.04±0.06	23.26±0.03	2.5±0.27
S292	*Salmonella* Enteritidis	0.28±0.01	0.36±0.01	0.74±0.02	0.80±0.02	12.36±0.01	3.0±0.0
S300	Other *Salmonella* sp.	0.31±0.01	0.38±0.01	0.83±0.02	0.85±0.02	9.17±0.01	2.5±0.0
S312	Other *Salmonella* sp.	0.33±0.03	0.35±0.01	0.89±0.05	0.78±0.02	12.03±0.01	3.0±0.0
S320	*Salmonella* Enteritidis	0.30±0.02	0.34±0.01	0.80±0.04	0.75±0.03	11.15±0.01	2.0±0.0
S333	Other *Salmonella* sp.	0.25±0.01	0.31±0.01	0.65±0.02	0.68±0.04	10.43±0.01	2.0±0.0
S349	Other *Salmonella* sp.	0.41±0.02	0.49±0.02	1.13±0.04	1.12±0.05	22.45±0.02	2.0±0.0
S357	*Salmonella* Typhimurium	0.55±0.03	0.61±0.04	1.54±0.06	1.41±0.07	23.56±0.03	2.5±0.27
S368	Other *Salmonella* sp.	0.27±0.01	0.32±0.02	0.71±0.02	0.70±0.05	15.06±0.01	2.0±0.0
S374	Other *Salmonella* sp.	0.26±0.01	0.29±0.06	0.68±0.02	0.63±0.13	13.12±0.01	2.0±0.0
S386	*Salmonella* Enteritidis	0.31±0.03	0.36±0.03	0.83±0.06	0.80±0.08	10.25±0.01	4.0±0.0
S390	Other *Salmonella* sp.	0.30±0.02	0.42±0.01	0.80±0.04	0.95±0.03	15.41±0.01	2.0±0.0
S402	Other *Salmonella* sp.	0.39±0.01	0.50±0.01	1.07±0.03	1.14±0.02	21.55±0.02	2.0±0.0
S419	Other *Salmonella* sp.	0.37±0.01	0.46±0.02	1.01±0.04	1.04±0.05	20.27±0.01	3.0±0.0
S427	*Salmonella* Typhimurium	0.49±0.02	0.58±0.02	1.36±0.05	1.34±0.03	23.46±0.02	2.0±0.0
S439	Other *Salmonella* sp.	0.30±0.03	0.39±0.01	0.80±0.05	0.87±0.04	17.52±0.01	3.0±0.0
S444	Other *Salmonella* sp.	0.25±0.02	0.32±0.02	0.65±0.03	0.70±0.03	16.79±0.01	2.5±0.0
S456	Other *Salmonella* sp.	0.40±0.01	0.57±0.02	1.09±0.03	1.31±0.06	21.54±0.02	2.5±0.27
S463	*Salmonella* Enteritidis	0.33±0.01	0.38±0.03	0.89±0.04	0.85±0.05	11.07±0.01	2.0±0.0
S472	Other *Salmonella* sp.	0.34±0.06	0.41±0.16	0.92±0.13	0.92±0.30	17.43±0.01	2.0±0.0
S489	*Salmonella* Enteritidis	0.42±0.04	0.49±0.01	1.16±0.11	1.12±0.04	20.36±0.01	2.5±0.27
S493	Other *Salmonella* sp.	0.43±0.01	0.51±0.02	1.19±0.02	1.17±0.06	23.27±0.02	3.0±0.0

### Autoaggregation and coaggregation index of selected biofilm forming *Salmonella* species

**Fig 2:**
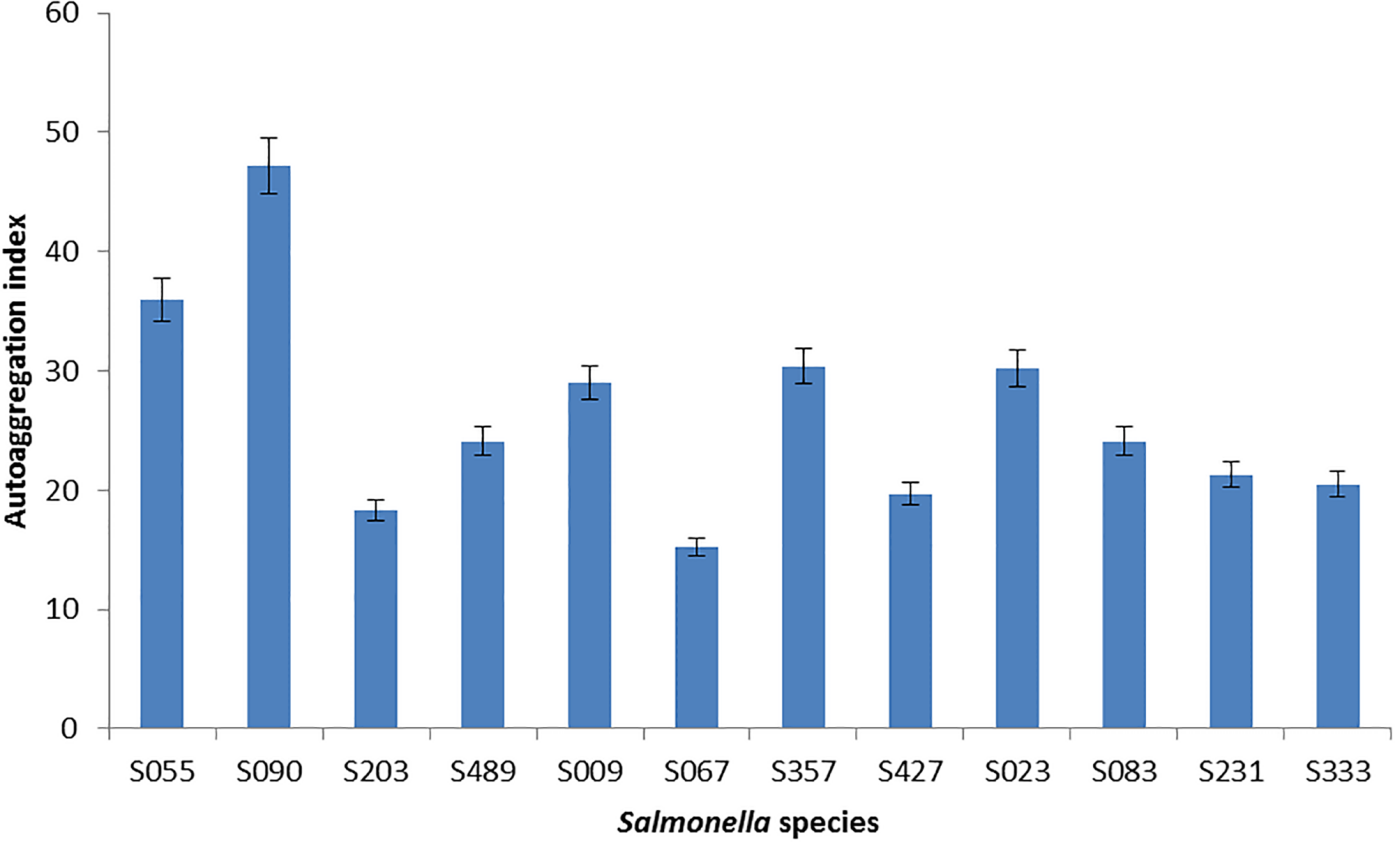
Autoaggregation index of selected *Salmonella* species. **S055**: *Salmonella* Enteritidis, **S09**: *Salmonella* Enteritidis, **S203**: *Salmonella* Enteritidis, **S489**: *Salmonella* Enteritidis, **S009**: *Salmonella* Typhimurium, **S067**: *Salmonella* Typhimurium, **S357**: *Salmonella* Typhimurium, **S427**: *Salmonella* Typhimurium, **S023**: Other *Salmonella* sp., **S083**: Other *Salmonella* sp., **S231**: Other *Salmonella* sp., **S333**: Other *Salmonella* sp.

**Fig 3:**
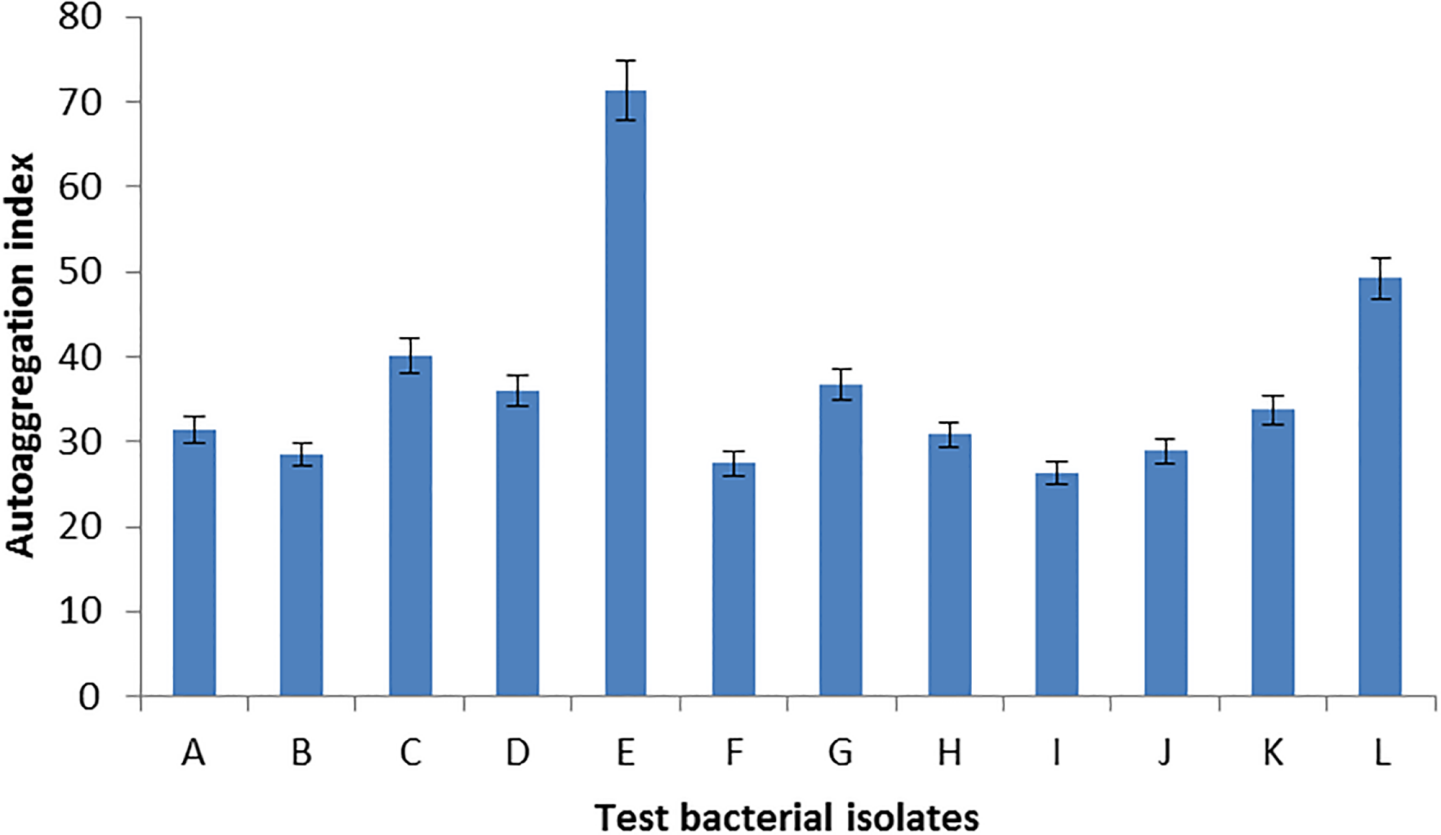
Autoaggregation index of selected test bacteria. **A**: *Aeromonas hydrophila* ATCC 7966, **B**: *Salmonella* Enteritidis ATCC 13076, **C**: *Acinetobacter baumannii* ATCC 19606, **D**: *Salmonella enterica* serovar Typhymurium ATCC 14028, **E**: *Staphylococcus aureus* ATCC 25823, **F**: *Escherichia coli* 29214, **G**: *Shigella flexnerium* ATCC 120222, **H**: *Listeria monocytogenes* ATCC 7644, **I**: *Listeria innocua* ATCC 33090, **J**: *Pseudomonas aeruginosa* ATCC 29853, **K**: *Pseudomonas putida* ATCC 15175, **L**: *Bacillus cereus* ATCC 14579.

A total of 5 moderate biofilm producers and 7 strong biofilm producers totalling 12 were assessed for their capacity to autoaggregate and coaggregate with other 12 test bacterial isolates. Autoaggregation index for the 12 selected *Salmonella* isolates ranged from 15.2–47.2% (Fig 2), while the autoaggragation index for the 12 selected test bacteria ranged from 26.2–71.3% (Fig 3). Coaggragation between the 12 selected test bacteria and 12 *Salmonella* isolates ranged from 12.5–81.0% (Table 5).

**Table 5:** Coaggregation index of selected biofilm forming *Salmonella* species.

Coaggregation indices (%)													
*Salmonella* isolates		S055	S090	S203	S489	S009	S067	S357	S427	S023	S083	S231	S333
Biofilm phenotype		++	++	+++	+++	++	++	+++	+++	+++	+++	+++	++
Partner strains	Range (%)												
*Aeromonas hydrophila* ATCC 7966	20.3–36.7	32.3	29.0	23.4	21.6	29.5	20.3	21.5	22.8	36.7	31.1	23.6	20.6
*Salmonella* Enteritidis ATCC 13076	19.0–35.1	23.5	22.7	31.2	28.5	34.2	35.1	19.0	24.6	23.5	29.6	23.2	22.4
*Acinetobacter baumannii* ATCC 19606	20.0–49.8	25.6	31.2	30.5	23.6	36.7	26.5	41.2	33.6	49.8	26.3	20.0	34.9
*Salmonella enterica* serovar Typhymurium ATCC 14028	23.6–45.0	25.7	34.2	40.5	24.6	42.1	34.6	37.8	24.5	39.6	32.1	45.0	23.6
*Staphylococcus aureus* ATCC 25823	23.4–81.0	23.4	35.6	65.2	34.8	32.5	56.3	81.0	65.7	34.3	28.5	45.3	49.0
*Escherichia coli* ATCC 29214	12.5–34.6	14.6	21.5	19.0	17.3	19.2	12.5	28.5	34.6	23.5	20.0	21.4	20.2
*Shigella flexneri* ATCC 120222	18.5–42.1	21.5	18.6	42.1	37.8	23.5	21.7	20.3	21.5	19.0	18.5	24.5	21.5
*Listeria monocytogenes* ATCC 7644	18.8–39.0	22.6	20.1	34.6	39.0	21.5	19.6	19.2	23.5	27.3	18.8	24.9	20.0
*Listeria innocua* ATCC 33090	20.4–32.3	25.3	27.1	22.5	21.3	29.5	23.6	21.4	29.5	32.3	26.7	21.6	20.4
*Pseudomonas aeruginosa* ATCC 29853	21.2–36.7	21.2	36.7	25.1	28.9	32.4	27.6	30.8	24.6	27.1	22.3	31.5	24.9
*Pseudomonas putida* ATCC 15175	21.4–43.6	31.5	25.6	39.4	23.6	32.7	43.6	25.0	21.5	32.4	39.0	21.4	31.3
*Bacillus cereus* ATCC 14579	21.5–56.3	31.5	23.6	21.5	26.7	32.4	52.5	56.3	50.1	29.5	32.6	27.8	31.5

**+++**: strong biofilm producers, **++**: moderate biofilm producers; **S055**: *Salmonella* Enteritidis, **S09**: *Salmonella* Enteritidis, **S203**: *Salmonella* Enteritidis, **S489**: *Salmonella* Enteritidis, **S009**: *Salmonella* Typhimurium, **S067**: *Salmonella* Typhimurium, **S357**: *Salmonella* Typhimurium, **S427**: *Salmonella* Typhimurium, **S023**: Other *Salmonella* sp., **S083**: Other *Salmonella* sp., **S231**: Other *Salmonella* sp., **S333**: Other *Salmonella* sp.

## Discussion

The disease causing capacity of *Salmonella* depends primarily on its virulence potential controlled by plasmid-borne or chromosomal determinants. The present study has elucidated the virulence factors, biofilm potential and cell surface characteristics of *Salmonella* species isolated from ready-to-eat shrimps. *Salmonella* remains one of the principal enteric foodborne pathogenic bacteria. Host-adapted species are able to initiate systemic infections to humans and strive for long periods of time, therefore posing significant problems of public-health [38]. *Salmonella* strains that are non-typhoidal are delineated by serological characterization into >2500 serovars of which the serovars Enteritidis and Typhimurium are the most prevalent [39–40]. While *Salmonella* species are passing through different host, natural and non-natural environments, they strives via diverse unfavourable environmental conditions, which includes nutrient availability, temperature fluctuations, presence of preservatives, changes in osmolarity, reactive nitrogen/oxygen species and antimicrobial peptides [5,41]. These diverse conditions influence different areas of its cellular physiology, such as growth, antimicrobial resistance and virulence.

Investigation in the present study showed that the *Salmonella* species from ready-to-eat shrimps had virulence potentials particularly strains of *Salmonella enterica*. These virulence potentials include the presence of protease, haemolysis, gelatinase, S-layer, swimming and swarming motility and DNA degrading activity. A positive correlation existed between *Salmonella* swarming motility and biofilm formation in the present study. However, scientific literature has reported that bacteria can ease their survival and proliferation by forming multicellular, cooperative communities which are frequently associated with surfaces [42]. These organized densities of microorganisms have been reported in environmental and clinical settings, where they impact negatively on microbial ecology and human health [43]. As such, the multicellular nature of bacterial is extensively studied; with biofilms being the most frequently recognised to this type of characteristics [1]. Swarming has been reported as another principal pattern of a surface-associated united process of bacteria. Swarming accelerates the prompt colonization of surfaces via micro-colonies of bacteria as a means of migration [44]. Bacterial swarming motility has been positively correlated with virulence factor production, antibiotic resistance and biofilm formation [45–46]. Most virulence factors known to be accompanied with swarming occurrences are more specifically extracellular proteases and exoenzymes [42]. A significant linkage that exists between extracellular protease production, pathogenesis, and swarming has been reported previously [47]. There appear to be several important factors in stimulating a swarming phenotype and they include viscosity of the medium, nutrient content and cell density. This result in a condition where cells become elongated and hyperflagellated coupled with the establishment of groups of cells via cell-to-cell contacts, which finally migrates as microcolonies in response to the factors that stimulates swarming phenotype [42].

Proteases are significant class of biomolecules that cleaves peptide bonds. They occur in all biotic life forms where they exhibit lots of important functions physiologically such as widespread degradation of protein to a more precise regulatory activity [48]. Extracellular proteases can breakdown both non-self and self-molecules with identical efficiency and less substrate selective recognition [48–49]. Extracellular proteases are stimulated in a multifaceted cascade that involves proteolytic maturation and auto-processing [48]. Previous literatures which reported that biofilm matrix is composed chiefly of polysaccharides, has been refuted with recent literatures on extracellular DNA (eDNA) and surface proteins as significant factors in the formation of biofilm, its regulation and stability [50–51]. A significant negative correlation of protease on biofilm formation was observed in this study which corresponds with recent reports and revealed that the function of proteases becomes clearer where the application of proteases of different origin to bacterial cultures have negatively correlated biofilm formation and dispersal of already formed biofilms [49,52]. Extracellular proteins portray diverse functions in biofilm, partaking in quorum-sensing functions and structure coupled with extracellular enzymes operating within the matrix [53].

Extracellular DNA presence in the structural matrix of multicellular origin has been elucidated to influence the biofilm structure and/or primary attachment of different species of bacteria [54–56]. The occurrence of extracellular DNA in nature seems to be ascribed with both active secretion and lysis of cells. Within the marine milieu, extracellular DNA is crucial in the ecosystem as a phosphorus and nitrogen reservoir [57]. With *Salmonella* being an autochthonous bacterial explains why such high percentage of extracellular DNA degrading activity was observed. The occurrence of extracellular DNA could be as a consequence of either vesicle release [58] or cell lysis [55–56], with active transport been a significantly speculative explanation. The role of extracellular DNA in biofilm structure includes a role as energy, nutrition source and structural component, or a gene pool for horizontal gene dissemination. A study by Harmsen et al. [59], revealed that when a short DNA fragment (< 500 bp) was incorporated into an extracellular DNA-free culture before adding salmon sperm or genomic DNA, adhesion was circumvented, portraying that high-molecular-weight DNA is requisite for adhesion and that the quantity of adherent sites on the cell surface can be saturated. In recent years some literatures have revealed the role played by multidrug efflux pumps in the capacity of *Salmonella* spp. to form biofilm [60]. Therefore, biofilm production, fitness and resistance seem to be interrelated [61]. Extracellular DNA has been reported to impede the development of biofilm by *Salmonella* Typhimurium and *Salmonella* Typhi on abiotic surfaces [62].

Gelatinase are reported to be the enzyme that is most studied of the clan of matrix metalloproteinases which contains stromelysins, membrane-type MMPs, collagenases and metrilysins. Gelatinase are proteolytic enzymes that allows living organisms to breakdown gelatin into compounds (amino acids, peptides and polypeptides) that crosses the cell membrane and facilitates cell migration into host cell. Arriving through the quorum-sensing system, gelatinase has been reported to being intricate in the formation of biofilm, via signal mediation [63] and translocation of bacteria across intestinal cell layers [64] which explain why a positive correlation existed between the gelatinase production and the formation of biofilm.

Haemolysis on blood agar by *Salmonella* species refers to proteins and lipids breakdown in red blood cells resulting in the release of haemoglobin thereby destroying their cell membrane. One mechanism where hemolysin lyses red blood cells is by pores formation in phospholipid bilayers [65]. Other hemolysin destroys erythrocytes by breaking down the phospholipids in the bilayer. There was no correlation between haemolysin from the *Salmonella* isolates and biofilm formation in this study. Hemolysin is a potent virulence factor which can put a human’s health at risk. The fact that hemolysins are combined with other virulence factors can to a greater extent threaten a human’s life. The significant consequence of hemolysis is hemolytic anemia.

A significant positive correlation exists between lipolytic activity and biofilm formation. Inactivation of the *lip*C gene significantly resulted in impaired type IV pilus-dependent swarming and twitching motility, as well as flagella-mediated swimming motility, with *lip*C mutant significantly portraying altered biofilm architecture [66]. Some species of *Salmonella* produce lipases which breakdown esters of glycerol with preference to fatty acids long-chained. This occurs at the generated interface in a hydrophilic aqueous medium through a hydrophobic lipid substrate [67]. A distinctive characteristic of lipases is referred to as interfacial activation which presents to the enzyme an interfacial area. With few exceptions, bacterial lipases are completely able to cleave a triacylglycerol substrate though certain preference for principal ester bonds has been observed. Bacterial lipases are produced during the bacterial infections process *in vitro* and, extensively damage the functionality of differentiated cell types that are involved in human immune response such as platelets or macrophages. Lipases are important virulence factors which utilize their detrimental effects in conjunction with other extracellular enzymes, with phospholipases C in particular [68].

S-layer is a regularly ordered layer in the outermost cell envelop component of numerous archaea bacteria. The presence of S-layer was negatively correlated with the formation of biofilm in this study which corresponds a previous finding by Auger et al. [69]. Biological functions of S-layer include protection against bacteriophages and phagocytosis; resistance to low pH; barrier against lytic enzymes; provision of adhesion sites for exoproteins [70]. S-layer has been reported to play significant roles in adhering to surfaces as well as their involvement in cell surface hydrophobicity [71–72].

*Salmonella* species in this study revealed biofilm potential at different temperature regimen; nutrient variability as well as different incubation condition. This shows that *Salmonella* species have developed complex, multiple adaptation to stress condition in concomitance with previous literature [5, 73]. In addition, their survival strategies by forming biofilms make them incredibly versatile and adept pathogens. This successful adaptation via overlapping stress response networks could be accomplished by a complex and coordinated programme of protein activity and gene expression, involving an array of two component regulatory systems, sigma factors and transcriptional regulators [74]. Bridging the different stress responses into a multiple strategy for pathogenic potential allows the organism to persist, survive and adapt in abiotic and biotic environments [75–76]. The difference in biofilm formation potential of the *Salmonella* species could be ascribed to difference in nutrient (EAOB and TSB), incubation temperature (21, 30 and 37°C) as well as static and dynamic environs, coupled with species diversity as previously reported in literatures [5,73,77–78].

Cell surface hydrophobicity is a significant factor that influences bacterial adhesion. The molecular nature of bacterial cell surface is important in the interaction between the host and microorganisms. Generally, microbes have been observed to produce biofilms under certain conditions such as nutrients deficient areas, inhibitory agents to include toxins or antibiotics under threat conditions for survival and protection [79]. There was no correlation between bacterial hydrophobicity and biofilm formation in this study. Most of the isolates in this study were hydrophilic indicating the importance of cell surface hydrophobicity for bacterial adhesion. However, a study by Mazumder et al. [80] revealed that biofilm formation resulted in the alteration of hydrophobicity of surface substratum further suggesting that bacterial cells adhered to surfaces are capable of modifying that surface. Extracellular compounds and surface components of bacterial, particularly exopolysaccharides, lipopolysaccharides and flagella, in conjunction with quorum-sensing and environmental signals, play vital roles in biofilm development, autoaggregation, coaggregation, colonization and host survival [81]. The physiological and structural complexity of biofilms has revealed that they are cooperative and coordinated groups, equivalent to multicellular organisms [82]. This is categorized via co-expression of the extracellular matrix components "thin aggregative" (curli) cellulose and fimbriae. Curli are particularly involved in cell aggregation, biofilm formation, and adhesion to surfaces. Curli also mediate host cell invasion and adhesion, and are potent inducers of the host inflammatory response. The functions of many of these adhesins (such as lipopolysaccharides, flagella, capsular polysaccharides) are not always very well understood, however several studies have revealed their significant roles in biofilm formation, multicellular behaviour, and cell aggregation [83–85]. *Salmonella* species and test bacteria selected in this study revealed autoaggregation and coaggregation capacities respectively. Literatures have revealed that production of biofilm by *Salmonella* spp. may be enhanced by the presence of other bacteria [44,86]. Literatures have also revealed that the presence of other species may deter formation of biofilm by *Salmonella* spp. [87]. *Salmonella* can produce various cell surface structures particularly of carbohydrate and proteinaceous nature. This may result in well-organized coaggregation of its own strains with cells of other species, likewise with cells of the same species, easing the formation of either multi- or mono species communities of biofilm subject to the surrounding conditions. In addition, the effect of the concurrent occurrence of other bacteria on the capacity of *Salmonella* to produce biofilms varies greatly with respect to the bacteria tested and environmental conditions.

## Conclusion

The present study has characterized the biofilm formation capacity, potential extracellular virulence factors, and cell surface characteristics of *Salmonella* species from ready-to-eat shrimps. The occurrence of pathogenic species of *Salmonella* from ready-to-eat shrimps could be detrimental to the consumers. Findings on the physiological conditions of biofilms formed by the foodborne pathogenic *Salmonella* and the cell surface characteristics therein are crucial for the advancement of methods for controlling *Salmonella* infections in ready-to-eat seafood.
